# Rejection Mechanism of Ionic Solute Removal by Nanofiltration Membranes: An Overview

**DOI:** 10.3390/nano12030437

**Published:** 2022-01-27

**Authors:** Nur Syahirah Suhalim, Norherdawati Kasim, Ebrahim Mahmoudi, Intan Juliana Shamsudin, Abdul Wahab Mohammad, Fathiah Mohamed Zuki, Nor Laili-Azua Jamari

**Affiliations:** 1 Faculty of Defence Science and Technology, National Defence University of Malaysia, Kem Sungai Besi, Kuala Lumpur 57000, Malaysia; syahirahsuhalim@yahoo.com; 2 Department of Chemistry & Biology, Centre for Defence Foundation Studies, National Defence University of Malaysia, Kem Sungai Besi, Kuala Lumpur 57000, Malaysia; intanjuliana@upnm.edu.my (I.J.S.); azua@upnm.edu.my (N.L.-A.J.); 3 Department of Chemical and Process Engineering, Faculty of Engineering and Built Environment, Universiti Kebangsaan Malaysia, Bangi 43600, Selangor, Malaysia; mahmoudi.ebi@ukm.edu.my; 4 Centre for Sustainable Process Technology (CESPRO), Faculty of Engineering and Built Environment, Universiti Kebangsaan Malaysia, Bangi 43600, Selangor, Malaysia; drawm@ukm.edu.my; 5 Department of Chemical Engineering, Faculty of Engineering, University of Malaya, Kuala Lumpur 50603, Malaysia; fathiahmz@um.edu.my

**Keywords:** nanofiltration, size exclusion, Donnan exclusion, membrane separation

## Abstract

The toxicity of heavy metals can cause water pollution and has harmful effects on human health and the environment. Various methods are used to overcome this pressing issue and each method has its own advantages and disadvantages. Membrane filtration technology such as nanofiltration (NF) produces high quality water and has a very small footprint, which results in lower energy usage. Nanofiltration is a membrane-based separation technique based on the reverse osmosis separation process developed in the 1980s. NF membranes have a pore size of 1 nm and molecular weight cut off (MWCO) of 300 to 500 Da. The properties of NF membranes are unique since the surface charge of the membranes is dependent on the functional groups of the membrane. The rejection mechanism of NF membrane is unique as it is a combination of various rejection mechanisms such as steric hindrance, electric exclusion, dielectric effect, and hydration mechanism. However, these mechanisms have not been studied in-depth due to their complexity. There are also many factors contributing to the rejection of NF membrane. Many junior researchers would face difficulty in studying NF membrane. Therefore, this paper is designed for researchers new to the field, and will briefly review the rejection mechanisms of NF membrane by both sieving and non-sieving separation processes. This mini-review aims to provide new researchers with a general understanding of the concept of the separation process of charged membranes.

## 1. Introduction

**Figure 1:**
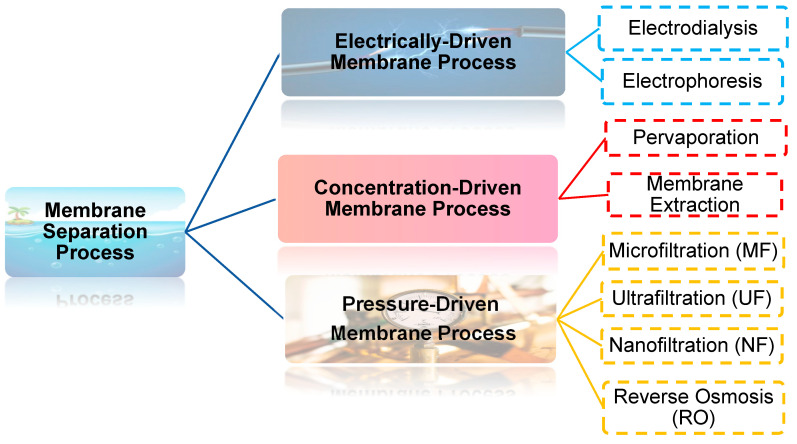
Classification of water treatment techniques.

Water pollution by heavy metals is known to be toxic and harmful to human health and the environment. As the human population grows, water pollution cannot be avoided due to humankind’s activities in various sectors, including industry, agriculture, and service. To evade this problem, several treatment technologies have been investigated by Crittenden et al. [1], such as reverse osmosis, disinfection, granular filtration, gravity separation, coagulation–flocculation, air stripping and aeration ion exchange, adsorption, and membrane filtration. Of these conventional treatments, membrane filtration technology has gained widespread popularity because of its numerous benefits in water treatment and its ability to achieve the required standard. Its benefits include space efficiency, remarkable energy reduction, and cost effectiveness [2]. In comparison, other conservative treatments are well-known for their ineffectiveness as well as production of hazardous products such as trichloromethane [3]. Furthermore, membrane filtration technology has the potential to close the gap between the economic and sustainability, environmental friendliness due to its less or no chemical requirement, and widespread accessibility [4]. As depicted in Figure 1, the membrane separation process is divided into three groups: the electrical-driven membrane process (electrodialysis and electrophoresis), the concentration-driven membrane process (pervaporation, membrane extraction), and the pressure-driven membrane process (microfiltration, ultrafiltration, nanofiltration, and reverse osmosis).

**Figure 2:**
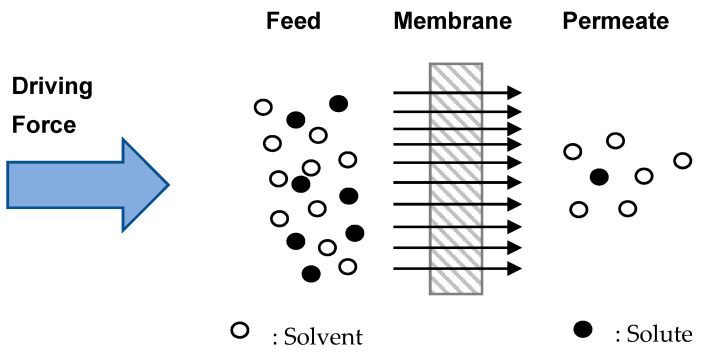
Schematic representation of the membrane filtration process.

Generally, in the membrane separation process, some substances are able to pass through (permeate) but some will be retained (retentate phase). According to Malliga et al. [5], the membrane filtration process (pressure-driven) is a method that uses membrane micropores to filter and employs selective permeability of membranes to separate particular substances in wastewater. A schematic representation of membrane filtration is displayed in Figure 2. As illustrated in Figure 2, the membrane acts as a barrier separating feed and permeate. The barriers considered here do not prevent the passage of all species but are permeable to some and impermeable to others. Since the number of species transported across a membrane is inversely related to its thickness, having the thinnest membrane feasible is favorable. Furthermore, pore aspect ratio and pore size distribution of the membrane also play an important role. It was found that increasing the pore aspect ratio improves membrane performance, while increasing the width of the pore size distribution deteriorates the performance [6]. Membrane performance is also dependent on process operating parameters such as feed concentration [7], feed flow rate, feed temperature, permeate vapor pressure [8] and applied pressure [9].

Each of these separation processes is distinctive since each of them works differently. The most critical factors in determining which separation process to use are efficiency and cost effectiveness [10]. Pirsaheb and colleagues took these two properties into account when comparing electrodialysis (ED) and reverse osmosis (RO) in order remove nitrate from drinking water. The results of their study showed that reverse osmosis is more efficient than electrodialysis in terms of cost per cubic meter of treated water and removal of nitrate and other chemical parameters. Therefore, their study distinguished that ED produces more waste energy than RO. The latest research [11] agreed with this conclusion; this research claimed that, in terms of capital cost, ED is less efficient than RO. It also asserts that estimates provided by industry showed that the area of normalized cost of ED is more than 200 $ m^−2^, while that of RO is less than 10 $ m^−2^. 

Table 1 summarises the distinctions between NF, UF, MF, and RO. The differences between these techniques were discovered through a series of studies conducted by Kei [12], Root [13], and Singh et al. [14]. As demonstrated in Table 1, NF is the most ideal for removing polyvalent anions, cations, uncharged compounds, and suspended particles. NF membrane is a relatively new technology in water and wastewater treatment [15]. Due to its unique rejection mechanism, many researchers have investigated NF membrane separation process. There are numerous additional advantages to NF membrane, which will be discussed in detail in the following section. Therefore, this paper will review the mechanism of nanofiltration membrane to assist readers in understanding these mechanisms.

## 2. Nanofiltration Membrane

Nanofiltration is a pressure-driven membrane-based separation technique developed in the 1980s based on the reverse osmosis separation process [16]. The pore size of NF membranes is 1 nm [17] and the molecular weight cut-off (MWCO) is 300–500 Da [18]. NF membrane is slightly charged due to dissociation of the functional group and adsorption of charged solute. It has been reported that the surface of NF polymeric membrane becomes slightly charged when in aqueous solution because of ionisable groups such as carboxylic groups and sulfonyl groups attached to the surface of the membrane [19]. Furthermore, the properties of NF membrane have high removal efficiency for divalent ions (e.g., calcium and tin) but poor rejection of monovalent ions [20]. NF membrane has other advantages, such as simplicity, durability, energy efficiency, and impurity removal [21]. NF membrane is also known for its high permeability, which is crucial for reducing energy consumption in water and wastewater treatment. Several researchers have developed a thin nanofiltration membrane by introducing an in situ formed interlayer into the TFC membrane by using the chitosan (CS)-assisted interfacial polymerization process [22]. Some researchers also have developed a highly permeable thin film NF membrane by using surface treatment with air–Ar plasma [23]. 

The disadvantage of NF membrane is the occurrence of membrane fouling. Membrane fouling is the accumulation and deposition of unwanted materials on the membrane’s surface and within its pores [24]. Membrane fouling is mainly dependent on the characteristics of sludge, operating parameters, membrane materials and configuration, and characteristics of feed water [25]. Membrane fouling is classified as either biofouling (agglomeration of microorganisms, plants, algae, or small animals) or organic fouling (accumulation of natural organic materials such as humic and fulvic acid). Membrane fouling can also be classified based on the degree of foulant removal to which some membrane fouling is reversible, although it is sometimes irreversible [26]. Reversible fouling can be eliminated through backwashing or intermittent operation of the membrane [27]. Irreversible fouling can be removed using chemical agents and bio-acid solutions such as lactic acid, propionic acid, and formic acid [28]. There are several methods for modifying the surface of NF membranes to achieve membrane fouling removal [29]. The methods available are UV photografting [30], plasma-assisted grafting [31], redox-initiated grafted polymerisation of acrylic acid [32] and embedding nanoparticles coupled with graphene oxide [33].

Other than suppressing membrane fouling, materials used in membrane fabrication could improve the cost efficiency and its rejection performance of nanofiltration. Selection of material for the membrane fabrication plays a vital role in the rejection mechanism [34]. This statement is consistent with the findings of Zhao et al. [35], who investigated the role of membrane and compound properties in determining the rejection of pharmaceuticals (PhACs) by various types of NF membranes (reverse osmosis membrane, tight NF membrane, and loose NF membrane). PhACs are known to contain trace organic compounds (TrOCs), and almost all TrOCs can physically or chemically interact with the membrane material. The interaction leads to adsorption on the membrane and consequently affects the rejection performance. In line with this statement, Schäfer and colleagues stated that there is a particular interaction between TrOCs and the functional group of polyamide and cellulose membrane due to hydrogen bonding. This functional group can act as proton donors or acceptors [36]. Therefore, it can be concluded that it is critical to thoroughly understand the rejection mechanism of NF membrane so that the membrane selection process is straightforward and the operational settings are optimized for a particular application, such as heavy metal removal from wastewater [37], separation of monosaccharides and monovalent salts in the biorefinery and food industry [38], water softening [39], and brackish water treatment [40]. The established rejection mechanism of NF membrane (both by sieving and non-sieving) are discussed in the next section. 

## 3. Sieving Mechanism: Size Exclusion (Steric Hindrance)

**Figure 3:**
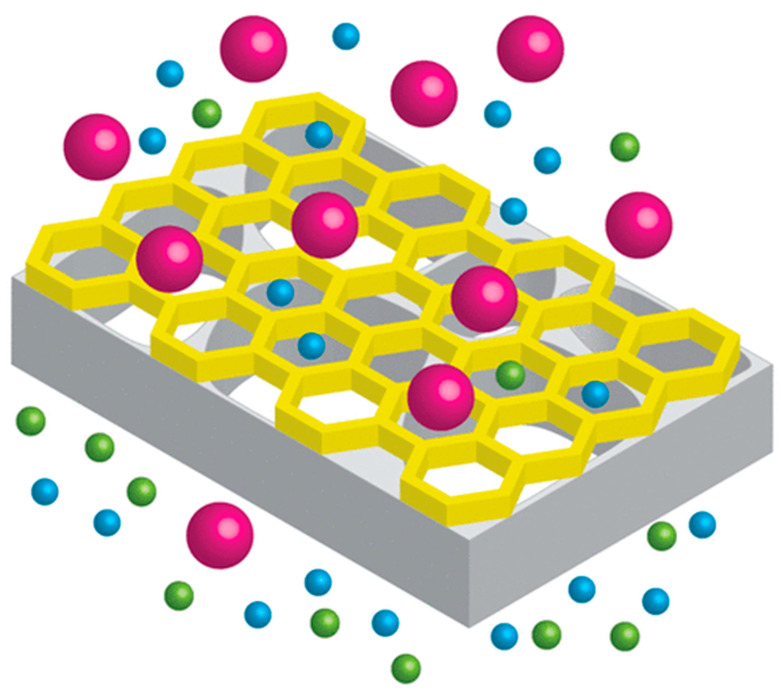
Steric hindrance effect. Reprinted with permission from Valentino, L.; Matsumoto, M.; Dichtel, W.R.; Marinas, B.J., 2017. Copyright Year (2017) Copyright American Chemical Society [45].

Sieving is a particle removal process that prevents a particle from passing through any pore or passageway smaller than the particle itself [41]. This process is illustrated in Figure 3. A particle that is smaller than the pore size can only pass through the membrane. Steric hindrance has been observed at the rejection of salts ions with a hydrated ionic diameter larger than the pore size of the membrane [42]. When the membrane separates neutral or uncharged solutes, the separation is primarily governed by steric hindrance, more commonly referred to as size exclusion. The rejection of As (III) at pH value 4.5–8.5 was governed by the size exclusion mechanism because As (III) remained uncharged at this pH range [43]. Meanwhile, Donnan exclusion controlled the rejection of As (IV), a mechanism which will be discussed in detail in next section. L. Zhu et al. [44] investigated the rejection of polycyclic aromatic hydrocarbons (PAHs) and phthalic acid esters (PAEs) as organic micropollutants. The results obtained from the study showed that the rejection of organic micropollutants was governed by steric exclusion alone since the organic micropollutants did not dissociate at pH value 7. Consequently, there was no ionic charge interaction between the trace organic micropollutants and the membrane. 

Numerous researchers have attempted to explain the effect of size exclusion during the separation process by developing an easy and effective method for elucidating the molecular characteristics (e.g., shape, dimension and molecular weight) of a molecule [46,47,48,49]. The results obtained from these various studies showed molecular weight (MW) of a non-charged compound could be a valuable parameter to predict rejection. However, investigation of how size exclusion affects the rejection mechanism is impossible due to insufficient information provided by MW about the geometry of a molecule. Therefore, as stated by Chang et al. [50], molecular size parameters such as molecular length, Stokes radii, and mean molecular size are better indicators than MW. As mentioned previously, if the solutes are neutral or uncharged, steric hindrance will be present. Some researchers have reported that neutral or uncharged solute filtrations are primarily used to characterize membranes [51]. Hence, for this purpose, a transport model is used. Vrijenhoek and Waypa [52] used the Spiegler–Kedem (SK) model to determine the pore size of the membrane by measuring the rejection measurement of saccharides. Others have used the steric hindrance pore model (SHP) to express the reflection coefficient [53]. Reflection coefficient is a measurement to describe the ability of a membrane in sieving a solute during the filtration process. 

One of the drawbacks of this model is that it ignores the concentration polarization (CP) effect, which is crucial in membrane designing. CP is the process of accumulation of retained solutes in the membrane boundary layer and creation of a high solute concentration at the membrane surface in comparison to the bulk solution. The concentration in the boundary layer is critical for both fouling and retention. At the membrane surface, there is a laminar boundary layer called the Nernst-type layer. There is mass conservationthroughout the layer, which is described by the film theory model. In this film theory model, feed concentration, solute diffusivity, and solute concentration in the boundary layer and the distance from membrane are taken into consideration. Due to the drawback of the SK model, a corrected model was invented by coupling the SK model with the film theory and named the model the combined film theory–Spiegler–Kedem (CFSK) model [54]. The researchers also used CFSK to predict solute rejection in the treatment of industrial ultra-high-temperature (UHT) condensates by reverse osmosis. Velicangil and Howell [55] evaluated the steric properties of UF membranes by using the orifice model with rejection curves of three protein solutions (papain, bovine serum albumin (BSA), and ovalbumin). Numerous studies have recently used the Nernst–Planck model to investigate the steric effect between uncharged solutes [56]. One of the most notable findings of these studies is that their outcomes are not consistent because different uncharged solutes lead to varied estimations of mean pore radius (≈1 nm).

The advantage of this mechanism is that it is easy to understand since it separates solutes based on size. However, since NF membrane is exhibited bysmall pores, the interaction between solutes (mainly ionic solutes) and membrane cannot be governed by steric hindrance alone. Dependence on steric hindrance can lead to poor rejection of two different solutes that have similar sizes. NF membranes have pores that can reject unwanted solutes (retentate) while allowing wanted solute (permeate) to pass through the pores even though those two solutes have the same size. The separation of ionic species by NF membranes strongly depends on the membrane charge and pore size [43]. Therefore, knowledge of the surface and pore characteristics of NF membranes is essential as it can allow for predictions of membrane separation behavior. Since the sieving rejection mechanism is relatively easy to understand, this review will focus more on the non-sieving mechanism.

## 4. Non-Sieving Mechanism

Non-sieving mechanism is a particle removal process in which particles smaller than the membrane pores are captured by adsorption at the pore surface. NF membrane is considered unique as it lies between non-porous RO membranes (where transport is governed by a solution-diffusion mechanism) and porous UF membranes (where separation occurs as a result of size exclusion and charge effects) [15]. The non-sieving mechanism plays an important role for charged solute. As mentioned earlier, the Nernst–Planck model has been used to investigate the steric effect between uncharged solutes. Geraldes and Alves introduced NanoFiltran, an open-source program for modeling transport of multi-ionic solutions through NF membranes based on the extended Nernst–Planck equation [57]. The results show that NanoFiltran is a valuable tool for accurate and robust prediction of the mass transfer in nanofiltration of multi-ionic solutions. Next, Epsztein et al. [58] incorporated NanoFiltran in their work investigating the role of ionic charge density on Donnan exclusion (a non-sieving mechanism). A brief analysis of open-source codes has included in this paper as Supplementary Data.

**Figure 4:**
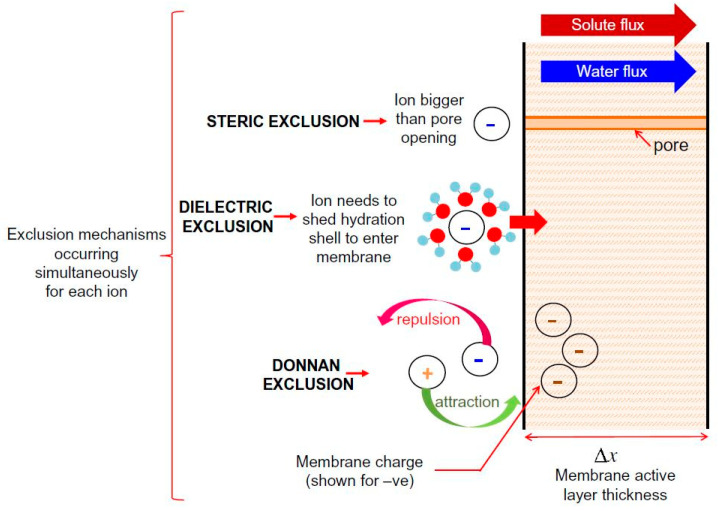
Schematic representation of solute exclusion mechanisms in nanofiltration as per the Donnan steric pore model with dielectric exclusion (DSPM–DE). Reprinted with permission from Yagnaseni Roy, David M. Warsinger, John H. Lienhard (2017). Copyright 2021 Copyright Elsevier [64].

It has been concluded that NF membrane has a complicated exclusion mechanism, which involves combining both sieving and non-sieving mechanisms. Supporting evidence of this statement can be found in various studies that have concluded that steric hindrance plays a predominant role in the rejection mechanism and that electrostatic effect also has a significant role during the separation process of TrOCs [59,60,61,62]. In addition, T. Fujioka et al. [63] compared the ceramic membrane and polymeric NF membrane by evaluating the rejection of TrOCs. The researchers determined that hydrophobicity influenced the interaction between TrOCs and the polymeric NF membrane during the rejection process, suggesting additional rejection mechanisms other than size exclusion. To the best of our knowledge, there are three accepted non-sieving mechanisms: Donnan exclusion, dielectric exclusion, and hydration mechanism. Donnan exclusion and dielectric exclusion mechanisms are illustrated in Figure 4. Figure 4 briefly explains both sieving (steric exclusion) and non-sieving mechanisms (Donnan exclusion and dielectric exclusion).

### 4.1. Donnan Exclusion 

**Figure 5:**
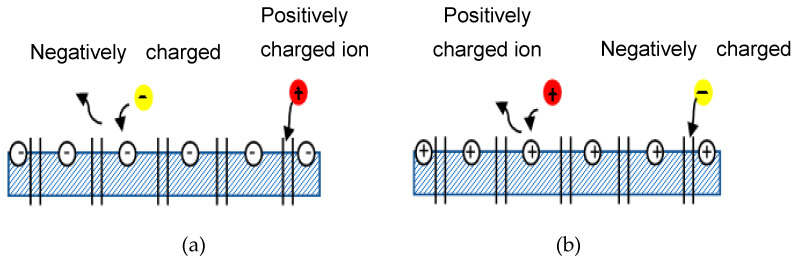
Schematic diagram of Donnan exclusion. (**a**) Negatively charged membrane, (**b**) Positively charged membrane.

Donnan exclusion occurs when ions partition into a material that contains high density of charge [65]. NF membrane is mainly governed by Donnan exclusion and size exclusion [29]. Emamjomeh et al. [66] also determined that the separation mechanism in NF membrane mainly involved the size of the molecules and the electrical response between the surface of the membrane and the ions in the feed. Furthermore, Donnan exclusion occurs due to electrostatic interactions between ionic solutes and the membrane matrix’s fixed electric charges [17]. Based on these claims from various researchers, one can conclude that NF membrane rejection is not solely determined by the size exclusion; it is also determined by Donnan exclusion. Figure 5 illustrates how Donnan exclusion works for both negatively and positively charged membranes. Negatively charged membranes reject negative ions (yellow ball) but allow positive ions (red ball) to pass through the membrane by transport of various solutes and entraps some of the ions in between the channel pore of the membrane. Solutes are transported by diffusion (movement of solute down a concentration gradient), by convection (solute transported by bulk fluid motion), and by electromigration (ion movement due to the membrane potential gradient [64]. The same law is depicted in reverse in Figure 5b.

To investigate the role of Donnan exclusion in the removal of arsenic by nanofiltration, Seidel et al. [43] evaluated surface charge, pore size, and rejection behaviour for salt solutions such as sodium chloride (NaCl), calcium chloride (CaCl_2_), and sodium sulphate (Na_2_SO_4_). They determined that the order of rejection rate for these salts is Na_2_SO_4_ > NaCl > CaCl_2_. These findings are consistent with Donnan exclusion. According to Donnan exclusion principle, the rejection of ions increases with co-ions that have higher charges and decreases with counter-ions that have higher charges. Na_2_SO_4_ has the highest rejection rate because it contains co-ions with the greatest negative charge, which is sulphate (SO_4_^2−^) ions. Simultaneously, CaCl_2_ has the lowest rejection since it has higher charge of counterions which is calcium (Ca^2+^). The rejection trends shown in their study demonstrate that the ion separation is governed by size exclusion and Donnan exclusion, as the observed trends do not correspond to the size of the hydrated ions.

**Figure 6:**
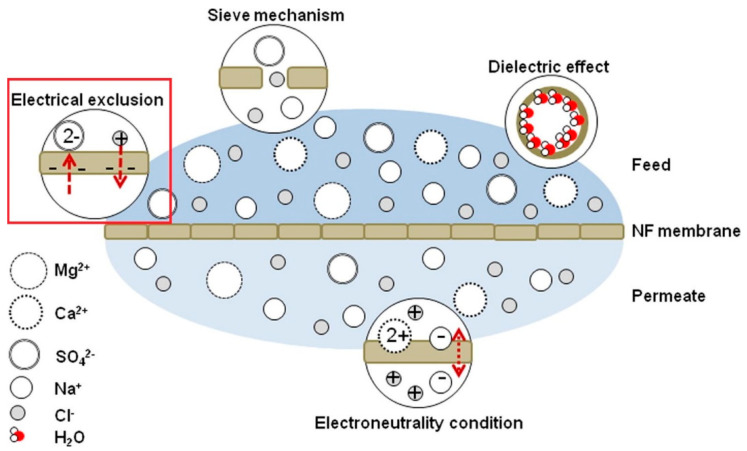
Illustrations of commonly involved rejection mechanism of NF membranes. Source: [64].

Donnan exclusion is also more pronounced on divalent ions [67,68]. Nicolini and colleagues evaluated the performance of NF membrane by measuring the saline rejection. The saline rejection for the NF membrane decreases in the following sequence: Na_2_SO_4_ > K_2_SO_4_ > CaSO_4_ > MgSO_4_ > NaCl. The saline rejection sequence is a result of anionic electrostatic repulsion and the preferred attraction of divalent cations. The red square shape in Figure 6 highlighted the Donnan exclusion mechanism where divalent anions such as sulphate ions (SO_4_^2^) are repelled while cations are attracted by the NF membrane. 

**Figure 7:**
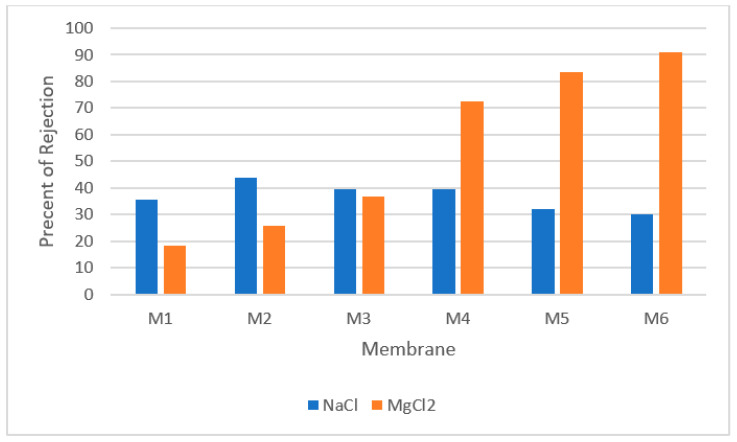
Concentration ratio of m-XDA and PEI [68].

Furthermore, the performance of fabricated polyamide (PA) NF membranes via interfacial polymerisation using m-xylylenediamine (m-XDA) and polyethyleneimine (PEI) as aqueous monomers were evaluated [68]. Liu and co-researchers controlled the concentration ratio of m-XDA and PEI from 1:0, 4:1, 3:2, 2:3, and 1:4 to 0:1 and named them M1, M2, M3, M4, M5, and M6. The researchers also investigated the selectivity of Na^+^ and Mg^2+^. The results showed that the addition of PEI to aqueous solution increased the rejection of NF membrane to magnesium chloride (MgCl_2_) and magnesium sulphate (MgSO_4_), but not to NaCl or Na_2_SO_4_. This is because both MgSO_4_ and MgCl_2_ have divalent cations (Mg^2+^), while NaCl and Na_2_SO_4_ only have monovalent cations (Na^+^). Therefore, Mg^2+^ rejection is determined by Donnan exclusion, whereas monovalent Na+ rejection is determined by steric hindrance (since Na^+^ has weak electrostatic repulsion). Figure 7 depicts the rejection of Na^+^ and Mg^2+^ by NF membranes. It shows that the rejection of Na^+^ in the M1–M6 membranes were not consistent since high PEI concentration contributed to low degree cross-linking and resulted in the reduction of steric hindrance. 

However, the Donnan exclusion mechanism is not sufficiently explained only by the rejection of ionic solutes. A model is needed to describe the rejection of ions by NF membranes. Consequently, a model to calculate ion rejection in single and mixed electrolyte solutions in RO and NF membranes has been proposed [69]. The researchers designed the model based on the extended Nernst–Plank equation and combined it with Teorell–Meyer–Sievers (TMS) model [70]. Bowen and Mukhtar proposed in 1996 that an ideal quantitative model for membrane processes should consider the physical properties of the membrane to predict separation behaviour under specified conditions [71]. The physical properties of the membranes that have been utilized are effective membrane thickness and charge density. They used a hybrid model (HM), which was initially developed for glomerular filtration and incorporated the extended Nernst–Planck equation and Donnan partitioning between the solution and membrane [72]. Next, in 1997, a new model called the Donnan steric pore model (DSPM) based on the hybrid model was developed by Bowen et al. [56]. This model has been widely used and evaluated because it describes Donnan exclusion sufficiently. For example, Donnan exclusion was used to predict the movement of ions (containing Ca^2+^, K^+^, and Cl^−^) through a negatively charged membrane. 

Schaep et al. evaluated the DSPM model using four commercial NF membranes to investigate the influences of molecular size, polarity, and charge on the retention of organic molecules. The researchers also evaluated the selected membrane with single-species salt solutions such as NaCl, Na_2_SO_4_, MgCl_2_, and MgSO_4_ [47]. The results showed that the membrane charge density was inconsistent and depended solely on salt type and concentration. Furthermore, due to divalent cations in the feed solution, the agreement between DSPM fitting and actual results was less than adequate. The model predicted an unreasonably high membrane charge density, and the presence of divalent cations changed the sign of the membrane charge density from negative to positive [73]. As a result, dielectric exclusion (DE) is used here as a secondary partitioning effect at the membrane-external solution interfaces, which will be discussed in greater detail in the following section.

### 4.2. Dielectric Exclusion 

**Figure 8:**
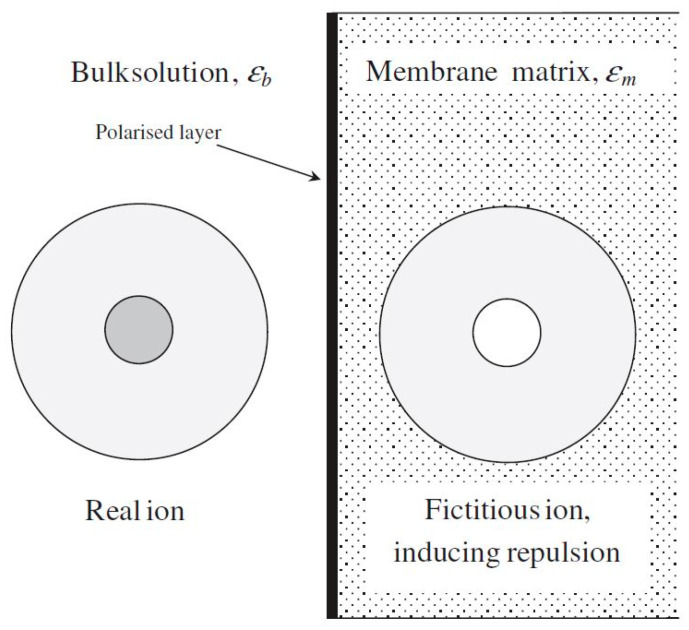
An ionic species approaching the NF membrane surface induces a repulsive image force. Reprinted with permission from Darren L. Oatley, Laia Llenas, Ramon Pérez, Paul M.Williams, Xavier Martínez-Lladó, Miquel Rovira (2012). Copyright 2021 Copyright Elsevier [75].

As mentioned in a previous section, it has been determined that there is an additional separation mechanism due to the unrealistic magnitude of membrane charge density predicted by DSPM. Therefore, Yaroshcuk et al. [74] introduced dielectric exclusion during the separation process by providing an in-depth analysis of this exclusion mechanism. The researchers stated that dielectric exclusion occurs when an ion interacts with the bound electrical charges generated by the ion at the interface of two materials with different dielectric constants: in this case, the membrane matrix and the solvent. It was determined that the ion polarizes the two media in accordance with their relative dielectric constants, forming a polarisation charge distribution at the discontinuity surface. This phenomenon is called production of image forces and is illustrated in Figure 8. It shows that if the membrane’s dielectric constant is less than the solvent’s, this image force will always be repulsive for both anions and cations. 

**Figure 9:**
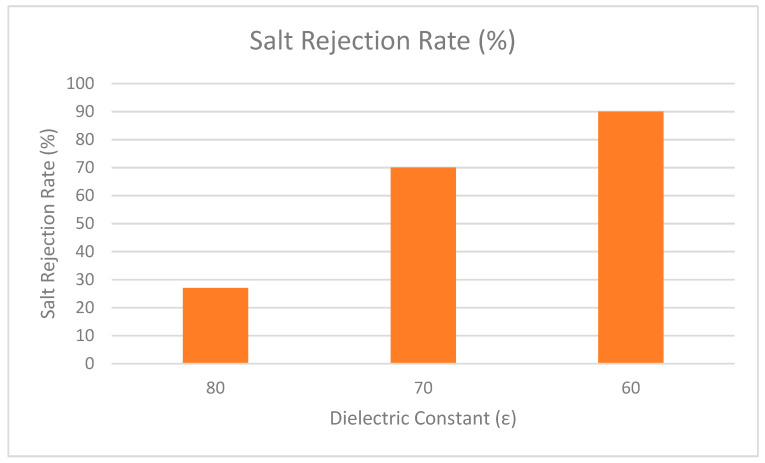
Rejection performance of salt at different dielectric constant.

In addition, Bowen and Welfoot widened the scope of the DSPM by incorporating more complex phenomena into their latest model [76]. Their study compared the effects of the solvent viscosity and the dielectric constant inside the pore. The results showed that variation of the solvent was insignificant, but the variation of the dielectric constant inside the pore was significant. It is believed that when the dielectric constant inside the pores decreases, it becomes smaller than the dielectric exclusion of bulk solution. Therefore, changes of the dielectric constant in the pores induced an excess energy barrier for ion solvation, preventing charged ions from partitioning into the pores. The effect was named dielectric exclusion (DE) and a Born equation was proposed to quantify it (Born effect). The researchers discovered that the dielectric effect reduced the charge density (extracted from experimental data) to a more reasonable number and improved model fitting when divalent salts were used. By incorporating the dielectric effect into the DSPM, this model is referred to as the Donnan steric pore model with dielectric exclusion (DSPM–DE) model. More recently, this model has been used widely by various researchers [77,78,79,80]. One of the highlighted studies explored the effect of dielectric constant in NF membrane [81]. The results showed that when the dielectric constant is decreased, the rejection performance is increased. As depicted in Figure 9, the dielectric constant was lowered from 80 to 60 and the rejection performance was increased as dielectric constant decreased. Therefore, it can be concluded that the dielectric constant holds an important role in the rejection performance of NF membrane. 

However, in practice, the DSPM–DE model is inconsistent because dielectric effects are based on slit-like pores, while the transport model is based on cylindrical pores. Later, Szymczyk and Fievet (2005) developed a steric, electric, and dielectric exclusion (SEDE) model. The researchers utilized both effects of dielectric exclusion mechanisms, which are the Born effect and image forces contribution [82]. The researchers stated that the SEDE model can be used to predict transport via cylindrical or slit-shaped pores. Later that year, Bouranene et al. [83] studied whether dielectric exclusion should be represented by the Born effect, the image force, or both. The modelling findings demonstrate that the experimental rejection rates are not adequately characterised when the two types of dielectric exclusion mechanisms are included. Hence, the researchers stated that the DSPM–DE developed by Bowen et al. (2002) was adequate for practical applications. Furthermore, as mentioned by Wang, researchers have made many modifications for various scenarios. However, the original DSPM–DE framework remains the most often utilized due to its capabilities of fitting experimental data in most cases [73].

### 4.3. Hydration Mechanism

As stated by Yaroshcuk, the hydration mechanism is not well understood [17]. It has been proposed that a loss of water dissolving capability of the membrane is likely caused by the changes to the dielectric properties of that particular membrane. The researcher also mentioned that multiple-charged ions should be considerably better excluded from the pores than single-charged ions which are sodium (Na^+^) and potassium ion (K^+^) [84]. Chen et al. concluded that the rejection of Na^+^ is higher even though K^+^ has a smaller hydration radius than Na^+^. This is because the diffusion coefficient of K^+^ calculated using Stokes’ law and Zwanzig’s theory is larger than that of Na^+^. Furthermore, the relationship between water molecules and hydrated cations passing through the graphene oxide (GO) membrane in a temperature-assisted system was studied [85]. Cha-umpong and others studied temperature-assisted systems as the driving force instead of pressure-assisted systems since theoretically, temperature as the driving force should not compromise salt rejection. 

**Figure 10:**
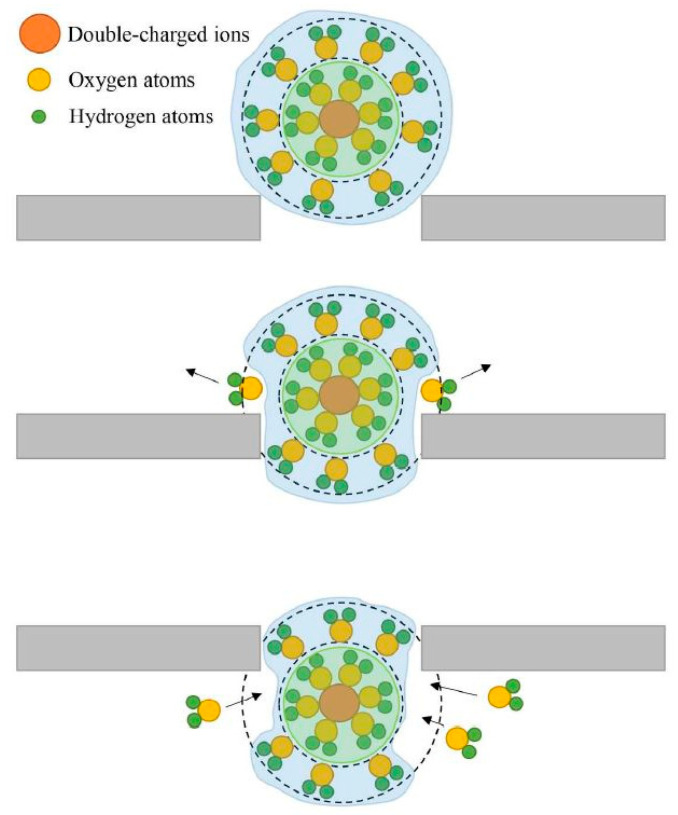
The schematic diagram of ions with a larger radius passing through the pore. Reprinted with permission from Chen, B.; Jiang, H.; Liu, X.; Hu (2017). Copyright 2017 Copyright American Chemical Society [84].

The researchers discussed why the rejection of Ca^2+^ is stronger than Mg^2+^ in terms of hydration radius. The first hydration radius of Ca^2+^ is bigger than that of Mg^2+^. The unique feature of divalent ions is their structure, as two layers of hydration shell surround the ions. The first layer is dense while the second layer (outer layer) is elastic, and this layer weakly connects with nuclear ions. If ions have a radius larger than the pore size, the ions can still pass through the pore because the water molecules adjust its outer hydration shell so that the inner hydration shell can pass through the pore. Figure 10 explains the mechanism of ions passing through the pore despite having a radius larger than the pore. Figure 10 displays several water molecules from the outer hydration shell being replaced, which allows the first hydration shell to easily pass through the pore. After passing through the pore, the second hydration shell attracts other water molecules in the permeated region to form a new hydration shell.

## 5. Donnan Exclusion as the Main Non-Sieving Rejection Mechanism

Nanofiltration membrane can be considered to have a negative charge on its surface. This was verified in a study done by Macnaughton et al. [86], who determined that most of the nanofiltration membranes are negatively charged at a neutral pH. The researchers stated that one of the factors that determined the surface membrane to be negatively charged or positively charged is the membrane functional group. Negatively charged membranes generally contain sulfonic acid groups (R−S(=O)_2_−OH), while positively charged membranes contain amine (−NH_2_) or imine groups. By changing the functional groups of the membrane, we can enhance the membrane selectivity. Hence, many researchers have devoted studies to fabricating NF membranes with tuning the charges on or in the selective layer to enhance the selectivity processes. Table 2 provides a summary of recent studies concerning the role of the surface charge of membrane. 

In Table 2, we can see that the rejection of salt for both negatively and positively charged membranes were mainly governed by Donnan exclusion mechanism. As discussed in Section 4.1, if the NF membrane is negatively charged, the rejection is increased for co-ions that have higher negative charge. Hence, the results for the rejection of salt by the negatively charged NF membrane in the constructed table were almost the same. The order of the rejection is Na_2_SO_4_ > MgSO_4_ > MgCl_2_ > CaCl_2_ > NaCl. The rejection of Na_2_SO_4_ is higher than that of MgSO_4_ even though both salts consist of sulphate (SO_4_^2−^) ions, which are the co-ions with the greatest negative charge. This is because Na_2_SO_4_ has the counter-ions with the lowest positive charge, sodium (Na^+^) ions, unlike MgSO_4_, which has magnesium (Mg^2+^) ions. The rejection is followed by MgCl_2_ since the salt consists of chloride (Cl^−^) ions. 

For the positively charged NF membrane, the order of the rejection of salt is MgCl_2_ > MgSO_4_ > NaCl > Na_2_SO_4_. As stated earlier, the principle of Donnan exclusion is that rejection increases if the salt consists of greatest charge of co-ions and smallest charge of counter ions. Since the membranes were positively charged, the co-ions were cations and the counter-ions were anions. Based on the order MgCl_2_ > MgSO_4_ > NaCl > Na_2_SO_4_, the rejection of MgCl_2_ was higher than that of MgSO_4_ since chloride (Cl^−^) ions are smaller than sulphate (SO_4_^2−^) ions. This principle also applies to NaCl and Na_2_SO_4_. Hence, it can be concluded that Donnan exclusion is the main non-sieving rejection mechanism in the charge status of the membranes.

## 6. Conclusions

Membrane technology is a rapidly growing, cost effective, low energy consumption, and environmentally friendly purification method for water and wastewater, which has significant progress and wide applications in the last decades. Aside from the advantages, membranes are best known for their unique rejection mechanism. The rejection mechanisms of the membranes depend not only on the size exclusion but also on other complex mechanisms such as Donnan exclusion and dielectric exclusion. If size exclusion is the only mechanism at play, the rejection of ions smaller than the pore size and larger than the pore is relatively low or almost zero. This article review is an in-depth look at Donnan exclusion, dielectric exclusion, and hydration mechanisms and the important role of these mechanisms in rejecting ions in different forms. However, studying the rejection process alone is clearly insufficient to investigate such complex and sophisticated rejection mechanisms. Therefore, these mechanisms cannot be determined only by measuring membrane permeability and solute rejections. There are further electrochemical and electrokinetic measurements that are critical factors for the determination of such mechanisms. Some models provide a more comprehensive understanding of the rejection behaviour of a membrane, namely the Spiegler–Kedem (SK), combined film theory–Spiegler–Kedem (CFSK), and Nernst–Planck models. These models can explain the characteristic rejection mechanism of the membranes. Finally, it cannot be denied that understanding the rejection mechanism of membranes is complex since few researchers have studied these mechanisms in depth. However, the authors believe that this mini review paper can explain the rejection mechanism of the membrane for researchers’ reference. The authors hope that this paper will be a stepping stone that can attract researchers’ interest, especially new researchers, in investigating in-depth the rejection mechanism and providing scientific explanations for the rejection mechanisms of the membranes.

## Figures and Tables

**Table 1:** Differences of RO, NF, UF, and MF based on pore size, operating pressure, components removed, and retain particulars.

	RO	NF	UF	MF
Pore Size (µm)	≥0.0001	≥0.001	≥0.01	≥0.1
Operating pressure (kPa)	1000–5000	500–1000	30–50	30–50
Components removed	almost all dissolved compounds and suspended particles	polyvalent anions, cations, uncharged compounds, suspended particles	high molecular weight compounds and suspended particles	ideally only suspended particles are removed
Retain particulars (MW)	<350	>150	1000–300,000	>300,000

Source: [10,11,12].

**Table 2:** List of published studies regarding NF membrane and their results for the rejection of salt.

Scope	Membrane Charged	Results	Refs.
NF-2012-250, polyamide thin-film composite membranes were used for the rejection of high divalent salt	Negative	CaSO4 > Na2SO4 > MgSO4 > MgCl2 > NaCl	[21]
Chitosan (CTS) and 1,3,5-triglycidyl isocyanurate (TGIC) were gradiently cross-linked on the polyethersulfone ultrafiltration membrane (PES)	Positive	MgCl_2_ > MgSO_4_ > NaCl > Na_2_SO_4_	[87]
Polyamide (PA) nanofiltration membranes with high solute-solute selectivity were prepared via a pre-diffusion interfacial polymerization (PDIP) process	Negative	Na_2_SO_4_ > MgSO_4_ > MgCl_2_ > CaCl_2_ > NaCl	[88]
Poly (vinyl alcohol) (PVA)/polydopamine (PDA) hybrid nanofiltration membrane was fabricated through aqueous electrospraying	Negative	Na_2_SO_4_ > MgSO_4_ > NaCl	[89]
A polyvinyl chloride (PVC)-based nanofiltration membrane was fabricated from polyvinyl chloride-*graft*-poly(*N*,*N*-dimethylaminoethyl methacrylate) by heating and crosslinking treatment	Positive	MgCl_2_ > CaCl_2_ > NaCl > MgSO_4_ > Na_2_SO_4_	[90]
Nanofiltration membranes were prepared by polydopamine (PDA) deposition followed by crosslinking on the polyethersulfone (PES) ultrafiltration (UF) membrane substrate.	Positive	MgCl_2_ > CaCl_2_ > NaCl > MgSO_4_ > Na_2_SO_4_	[91]
A membrane was fabricated via introducing 2, 5 diaminobenzenesulfonic acid (DABSA) into the polyamide layer	Negative	Na_2_SO_4_ > MgSO_4_ > NaCl > MgCl_2_	[92]
Polyamide (PA) thin film composite (TFC) membranes were prepared by interfacial polymerisation (IP) technique with trimesoyl chloride (TMC) and three acyl chloride groups	Negative	MgSO_4_ > Na_2_SO_4_ > MgCl_2_ > NaCl	[93]
Thermally cross-linked branched-polyethyleneimine (b-PEI) layer was introduced to a loose polyethersulfone NF membrane by dip-coating b-PEI and an epoxy linker and heat treatment in a sealed oven with a high-humidity atmosphere	Positive	MgCl_2_ *>* MgSO_4_ *>* NaCl *>* Na_2_SO_4_	[94]

## Data Availability

Not applicable.

## Supplementary Materials

The following supporting information can be downloaded at: https://www.mdpi.com/article/10.3390/nano12030437/s1.
