# Supplementary material for: Rejection Mechanism of Ionic Solute Removal by Nanofiltration Membranes: An Overview
Source: Nanomaterials (Basel). 2022 Jan 27;12(3):437. doi: 10.3390/nano12030437 (PMC8839881; doi:10.3390/nano12030437)
Supplement: Supplementary file 1 [file nanomaterials-12-00437-s001.zip › nanomaterials-1497293-supplementary.pdf]

nanomaterials

MDPI

Supplementary Data

Rejection Mechanism of Ionic Solute Removal by Nanofiltration Membranes: An Overview

Nur Syahirah Suhalim [1], Norherdawati Kasim [2,*], Ebrahim Mahmoudi [3], Intan Juliana Shamsudin [2], Abdul Wahab Mohammad [4], Fathiah Mohamed Zuki [5] and Nor Laili-Azua Jamari [2]

1 Faculty of Defence Science and Technology, National Defence University of Malaysia, Kem Sungai Besi, Kuala Lumpur 57000, W.P. Kuala Lumpur, Malaysia; syahirahsuhalim@yahoo.com
2 Department of Chemistry & Biology, Centre for Defence Foundation Studies, National Defence University of Malaysia, Kem Sungai Besi, Kuala Lumpur 57000, W.P. Kuala Lumpur, Malaysia; intanjuliana@upnm.edu.my (I.J.S.); azua@upnm.edu.my (N.L.-A.J.)
3 Department of Chemical and Process Engineering, Faculty of Engineering and Built Environment, Universiti Kebangsaan Malaysia, Bangi 43600, Selangor, Malaysia; mahmoudi.ebi@ukm.edu.my
4 Centre for Sustainable Process Technology (CESPRO), Faculty of Engineering and Built Environment, Universiti Kebangsaan Malaysia, Bangi 43600, Selangor, Malaysia; drawm@ukm.edu.my
5 Department of Chemical Engineering, Faculty of Engineering, University of Malaya, Kuala Lumpur 50603, W.P. Kuala Lumpur, Malaysia; fathiahmz@um.edu.my
* Correspondence: herdawati@upnm.edu.my

(1) Input the numerical parameters: number of discretization points, N, grid expansion factor, maximum number of iterations, normalized residuals target and the under-relaxation factor. If the grid expansion factor is higher than one, the distance between consecutive grid nodes expands by this factor from the membrane/solution interfaces to the center of the membrane active layer. If the mesh expansion factor is equal to one, the grid is uniform.
(2) Input the membrane pore radius, r_p, equivalent membrane thickness, Δx_e, membrane volume charge density, c_X and pore dielectric constant, ε_p.
(3) Input the operating parameters: permeate flux, J_v, temperature, T, number of ionic species, N_c, ions diffusivities, $D_{i,\infty}$, concentration of the ions in the feed-solution, $c_{i,b}$, parameters of the mass-transfer correlation, feed Reynolds number, and characteristic length of feed-channel, L.
(4) Initialize the variables $c_{i,m}$, $c_{i,j}$, $c_{i,p}$, Ψ_j, Ψ_p and ε The concentrations, $c_{i,m}$, $c_{i,j}$ and $c_{i,p}$, are initialized with the bulk concentration, $c_{i,b}$, and the initial values for Ψ_j, Ψ_p and ε are taken as zero.
(5) Compute the coefficients of the coupled linearized system of equations.
(6) Solve the previous linear system of equations using LU decomposition with partial pivoting. These calculations are accomplished using the subroutine SGEVS contained in the LAPACK package, which is a collection of Fortran subroutines for solving dense linear algebra problems.
(7) Under-relax the computed solutions.
(8) Update the coefficients of the coupled linearized system of equations using the under-relaxed variables.
(9) Compute the normalized residuals.
(10) Repeat the steps 5–9 until the normalized residuals are bellow a given target.
